# Iodine-Doped Hollow Carbon Nanocages without Templates Strategy for Boosting Zinc-Ion Storage by Nucleophilicity

**DOI:** 10.3390/ma17040838

**Published:** 2024-02-09

**Authors:** Ruiting Niu, Huailin Fan, Qingfu Ban, Dezhi Zhou, Lekang Zhao, Jiayuan Yu, Qifeng Chen, Xun Hu

**Affiliations:** 1 School of Material Science and Engineering, University of Jinan, Jinan 250022, China; 2 College of Chemistry and Chemical Engineering, Yantai University, 30 Qingquan Road, Yantai 264005, China; 3 Institute for Advanced Interdisciplinary Research (iAIR), School of Chemistry and Chemical Engineering, University of Jinan, Jinan 250022, China

**Keywords:** iodine doping, self-assembly, carbon nanocages, Zn-ion storage

## Abstract

Zn-ion hybrid supercapacitors (ZHCs) combining merits of battery-type and capacitive electrodes are considered to be a prospective candidate in energy storage systems. Tailor-made carbon cathodes with high zincophilicity and abundant physi/chemisorption sites are critical but it remains a great challenge to achieve both features by a sustainable means. Herein, a hydrogen-bonding interaction-guided self-assembly strategy is presented to prepare iodine-doped carbon nanocages without templates for boosting zinc-ion storage by nucleophilicity. The biomass ellagic acid contains extensional hydroxy and acyloxy groups with electron-donating ability, which interact with melamine and ammonium iodide to form organic supermolecules. The organic supermolecules further self-assemble into a nanocage-like structure with cavities under hydrothermal processes via hydrogen-bonding and π-π stacking. The carbon nanocages as ZHCs cathodes enable the high approachability of zincophilic sites and low ion migration resistance resulting from the interconnected conductive network and nanoscale architecture. The experimental analyses and theoretical simulations reveal the pivotal role of iodine dopants. The I_5_^−^/I_3_^−^ doping anions in carbon cathodes have a nucleophilicity to preferentially adsorb the Zn^2+^ cation by the formation of C^+^-I_5_^−^-Zn^2+^ and C^+^-I_3_^−^-Zn^2+^. Of these, the C^+^-I_3_^−^ shows stronger bonding with Zn^2+^ than C^+^-I_5_^−^. As a result, the iodine-doped carbon nanocages produced via this template-free strategy deliver a high capacity of 134.2 mAh/g at 1 A/g and a maximum energy and power density of 114.1 Wh/kg and 42.5 kW/kg.

## 1. Introduction

In recent years, electrochemical energy systems have increasingly drawn attention to storing intermittent and green energy (e.g., solar, tidal, and wind), due to their intrinsic environmental friendliness, high safety, and simple assembly process [1]. Among these, aqueous hybrid supercapacitors, by utilizing battery-type electrodes, capacitive-type electrodes, and aqueous electrolytes, are emerging as a potential energy storage device [2]. In terms of the design concept, zinc-ion hybrid capacitors (ZHCs) have drawn extensive attention because they combine the advantages of batteries and supercapacitors [3,4]. In view of Zn anodes with a low redox potential, superior theoretical capacity, massive production, and high safety to deliver sufficient charges during electrochemistry, the key is exploring high-performance carbon cathodes to coordinate Zn anodes for accomplishing the anticipated Zn^2+^ storage capability [5]. The carbon cathodes with various modifications, such as changing structure design, introducing heteroatoms, and increasing defects, were intensively investigated to enhance the number of ion adsorption sites and reduce ion transport paths in pore channels [4]. The perceptibility in this field is still in the initial stage of exploration, and the major problems confronted are deficient zincophilic capacity, and sluggish ion reactions of carbon cathodes, resulting in the less than expected energy density and high-rate sustainability [1]. As reported by many documents, the doping of heteroatoms (e. g., O, N, P, S, B, etc.) can regulate the wettability and electronic structure of carbon cathodes to boost the energy density [6,7,8,9]. The O and N-containing groups have pseudocapacitive reactions with the Zn^2+^ ion to form C-O-Zn and C-N-Zn bonds, which were close to the electron donor properties of O and N-containing groups [10]. In addition, the P and S functionalities promoted the O and N-containing groups reacted with Zn^2+^ ions to introduce additional pseudo-capacitance [10]. The iodide anion is also an electron donor, which possesses nucleophilicity similar to the O and N-containing groups. Thus, the iodide anion should also react with the Zn^2+^ ion to introduce additional pseudo-capacitance. Besides, the zinc–iodine batteries have shown a promising prospect in energy storage, which also demonstrated the bonding tendency between iodide and zinc [11]. Thus, iodide-doped carbon material is expected to be a key breakthrough in designing highly electroactive and stable cathode materials for ZHCs.

The rational design of carbon cathodes is another critical factor, which would affect the whole electrochemical index of ZHCs [12]. Among these, hollow carbon nanocages (HCNCs) are a kind of three-dimensional nanostructured carbon material consisting of curved carbon nanosheets with nanoscale voids, and the low-dimensional carbon nanosheets are constructed by graphene microcrystal as building units [13]. There are rich nanopores crossing the shells of carbon nanocages to expose the more accessible active sites [14]. Recently, the HCNCs with promising morphology and porous structures promoted the exchange and transfer of substances and effectively accommodated the strain relaxation in the energy storage reaction [15,16,17,18]. Currently, the synthesis strategies of HCNCs primarily depend on the template-based method, which faces a great deal of challenges such as low efficiency, and environmental concerns caused by etching agents [19]. Therefore, an innovative method to prepare HCNCs is expected, which would be an environmentally friendly and scalable approach suitable for practical applications.

The modular self-assembly method does not involve the templating application, and time-consuming steps, thereby enabling the facile and versatile preparation of HCNCs. Ellagic acid is an important component of plant extracts, which play a significant role in the field of biomedicine due to its antioxidant and chelator properties from OH groups [20]. Ellagic acid contains a core of fused aromatic rings, keeping a near planar molecular structure and extensional hydroxy and acyloxy groups [21]. The hydroxy and acyloxy groups as electron-donating groups boost the electron density of ellagic acid, endowing ellagic acid with the capability to participate in hydrogen bonding and π-π coactions. Under this feature, the concentration of melamine could be quantified by a strong hydrogen-bonding interaction to weaken the ellagic acid reducing ability [22,23]. Inspired by the strong hydrogen-bonding interaction, iodide-doped HCNCs were prepared by the modular self-assembly strategy without templates. The anticipated configurational features, such as hollow cage-like structure, high specific surface area, and abundant iodide-containing and oxygen-containing functional groups, for carbon cathodes were easily achieved. The specific cage-like morphology with cracking shells network structure effectively facilitated electrolyte diffusion as well as ion transfer during the charging/discharging process, leading to excellent rate capability and cyclability. The coadjacent shell structure boosted the electroconductibility, favoring rapid electron transport. In addition, the abundant specific surface area offered numerous active species for reversible ion adsorption and guaranteed the active sites approaching the electrolyte, resulting in decent charge storage capability. In addition, the iodine-containing and oxygen-containing groups displayed reversible chemisorption with Zn^2+^, providing additional pseudo-capacity. Integrating the availably of abundant storage sites, effective ion diffusion paths, and additional pseudo-capacitance, the obtained carbon nanocages showed an outstanding electrochemical performance as the cathode in ZHCs.

## 2. Materials and Methods

A total of 2 g melamine (Macklin, Shanghai, China), 0.5 g ammonium iodide (Macklin, Shanghai, China), and 2 g ellagic acid (Macklin, Shanghai, China) were put in 60 mL distilled water for stirring for 1 h. The mixtures were transferred into a Teflon-lined stainless-steel autoclave and kept at 180 °C for 16 h. The solid product was collected by filtration after it had cooled down to room temperature. After this, the collected hybrids were directly carbonized at 900 °C for 1 h under N_2_ atmosphere at a heating rate of 5 °C/min. The obtained carbon product was also further activated with potassium hydroxide (mass ratio: 1:1) at 900 °C for 1 h. The activated sample was washed with 0.5 M hydrochloric acid and dried at 70 °C, termed as I, N-CNCs. For comparison, 2 g melamine and 2 g ellagic acid were hydrothermally treated and activated as N-CNCs. CMs were prepared with 2 g ellagic acid under the same conditions as I, N-CNCs.

Morphological and structural characterization, electrochemical measurements, theoretical calculations, and computational formula were specified in supporting information.

## 3. Results and Discussion

### 3.1. Structure Characterizations

**Scheme 1:**
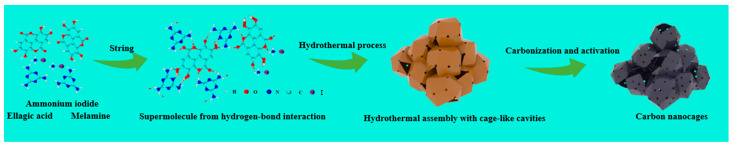
Schematic illustration of the preparation process of carbon nanocages.

The synthesis process of iodine and nitrogen-doped carbon nanocages basing on melamine, ellagic acid, and ammonium iodide was schematically displayed in Scheme 1. Ellagic acid contains a molecular structure comprising a planar biphenyl moiety bridged by two lactone rings and four hydrogen-bonding OH groups, which boost the electron density of ellagic acid as electron-donating groups, endowing ellagic acid with the capability to participate in hydrogen bonding and π-π coactions. Under this feature, melamine and ammonium iodide could interact with ellagic acid through a strong hydrogen-bonding interaction to form a supramolecular chelate owing to the strong electron-donating effect of ellagic acid and the electron-pulling ability of melamine and ammonium (Step I) [22]. The supramolecular chelate was further transferred into a nanohybrid solid material under a hydrothermal process (Step II). The TEM image of the hydrothermal product in Figure S1a showed the voids in the nanohybrids, which were possibly ascribed to the further self-assembly of supramolecular chelate from melamine and ellagic acid by the hydrogen-bonding interaction. Finally, carbon nanocages with well-maintained shape were prepared by carbonization and activization process (Step III). Correspondingly, the ruleless microparticles were observed in the product of ellagic acid and ammonium iodide under hydrothermal treatment as the contrast sample in Figure S1b. In addition, the connected voids in the hydrothermal nanohybrids from melamine and ellagic acid were displayed in Figure S1c. The energy dispersive spectroscopy (EDS) revealed that C, N, O, and I elements were uniformly distributed in the whole nanocage hybrids, verifying the successful self-assembly of supramolecular chelate from melamine, ellagic acid, and ammonium iodide into the nanocages structure (Figure S2). Thus, the melamine acted as the morphology-oriented reagent with the additional N source. The ellagic acid was used as a sustainable carbon source and ammonium iodide provided the doped iodine. The cavities were preserved during the carbonization and activation process and mastered the carbon morphology into hollow carbon nanocages consisting of curved carbon nanosheets with nanopores during the sublimation, decomposition and expansion of the organic inducer. The obtained iodine and nitrogen co-doped carbon nanocages were labeled as I, N-CNCs. The nitrogen co-doped carbon nanocages were denoted as N-CNCs under a similar process without ammonium iodide. In addition, the carbon materials without cage-like morphology were denoted as CMs.

**Figure 1:**
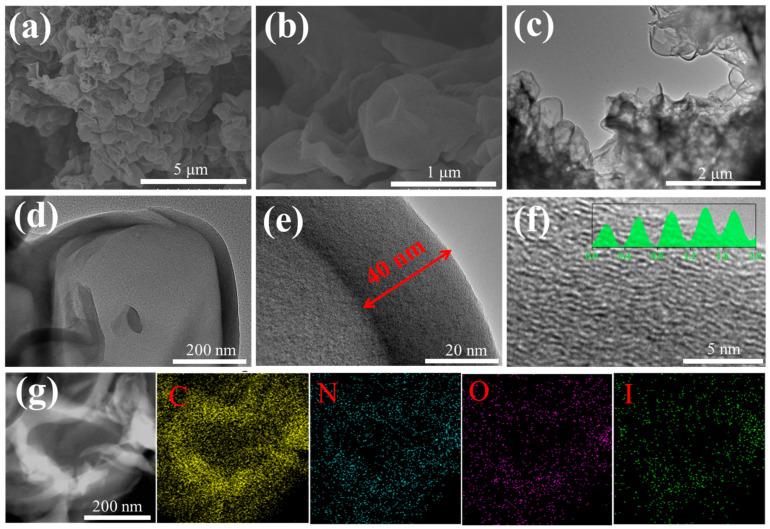
Electron microscopy characterization of I, N-CNCs. (**a**,**b**) SEM images of I, N-CNCs. (**c**–**e**) TEM images of I, N-CNCs. (**f**) High-resolution TEM image of I, N-CNCs. (**g**) HADDF-STEM image of I, N-CNCs and the corresponding elemental mapping images of C, N, O, and I.

The morphology and structure of the synthesized I, N-CNCs were revealed by SEM and TEM. As shown in Figure 1a, coadjacent carbon nanoparticles with a size of approximately 500 nm were observed. Among these, the cracked carbon shells displayed the formation of abundant cavities into nanoparticles. These cavities and carbon shells formed the nanocages-shaped carbon materials. The nanocages structure in Figure S1a was preserved after the carbonization and directed the further conversion of the hydrothermal hybrids into carbon nanocages during the decomposition of melamine. In addition, cage-like structures can stack with each other to form stacking holes. The building blocks in Figure 1b are the hollow nanocages structure with wrinkled and coarse shells, which was in favor of generating a large specific surface area and exposing abundant active sites. The TEM images (Figure 1c,d) confirmed the interconnected hollow carbon nanocages with cracked shells. The interconnected hollow and cracked carbon nanocages ensured a low transfer resistance and rapid charge transfers within the carbonaceous materials. Figure 1e demonstrates a uniform shell with a diameter size of approximately 40 nm. As shown in Figure 1f, the I, N-CNCs showed a defective configuration with local ordering, with a calculated layer spacing of 0.351 nm. In addition, elemental mapping revealed that that C, N, O, and I elements were uniformly distributed in the whole nanocages, verifying the successful doping of I, O, and N elements into the carbon nanocages (Figure 1g).

**Figure 2:**
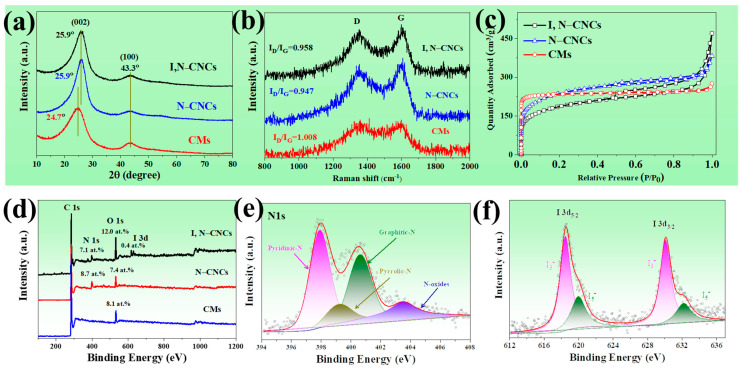
(**a**) XRD patterns, (**b**) Raman spectra, (**c**) nitrogen adsorption-desorption isotherms, and (**d**) XPS full spectra (data: relative content of elements) of I, N-CNCs, N-CNCs, and CMs, respectively. (**e**,**f**) N 1s and I 3d high-resolution XPS spectra of I, N-CNCs.

The crystal structure and graphitization degree of carbon materials samples were identified via XRD and Raman spectroscopy, respectively. Two distinctive broad peaks at 2θ of approximately 24.7° and 43.3° were observed for CMs in Figure 2a, which were ascribed to the (002) and (100) crystal planes of the graphitic carbon (JCPDF No.75–1621), respectively [24]. Obviously, the (002) peak with the narrower halfwidth for I, N-CNCs and N-CNCs shifted to the larger 2θ direction from 24.7° to 25.9° with the introduction of melamine, indicating more ordered (002) planes. For the I, N-CNCs, the interlayer spacing of the (002) plane was calculated to be 0.35 nm, and the crystal size along the *c* axis (*L*_c_), i.e., the thickness of the (002) plane was 1.6 nm (Table S1). The thickness of the (002) plane of I, N-CNCs was lower than that of N-CNCs, which implied that the iodine doping weakens the degree of graphitization. As shown in Figure 2b, two diffraction bands were displayed at around 1348 and 1587 cm^−1^, describing the random vibration of sp^3^ carbon species with defects and disorders (D band) and the in-plane vibration of sp^2^ hybridized carbons (G band), respectively. The ratio of the D and G band intensity (I_D_/I_G_) can assess the graphitization degree of samples [25]. The I_D_/I_G_ values of I, N-CNCs (0.958) and N-CNCs (0.947) were lower than that of CMs (1.008), indicating that CMs possessed more defects in the carbon skeleton, in keeping with XRD results.

N_2_ adsorption-desorption isotherms were applied to explore the porous characteristics of the carbon cathodes in Figure 2c, and the detailed data were listed in Table S2. At relatively low pressures (P/P_0_ < 0.01), the N_2_ adsorption capacity for all samples increased rapidly (type I for CMs and type IV for I, N-CNCs and N-CNCs), indicating the existence of a large quantity of micropores. The N_2_ adsorption/desorption isotherms of I, N-CNCs and N-CNCs exhibited another steep uptake (P/P_0_ > 0.97) and hysteresis loops (0.40 < P/P_0_ < 0.90), indicating the coexistence of mesopores and macropores. Thus, the CMs were dominated by micropores with a tiny proportion of mesopores and macropores. The S_BET_, V_total_, and V_micro_ of CMs were 944 m^2^/g, 0.42 cm^3^/g, and 0.35 cm^3^/g, respectively. The mesopores and macropores of N-CNCs with cage-like morphology increased significantly compared to those of CMs, and their V_total_ increased to 0.59 cm^3^/g. When the doping iodine by ammonium iodide was introduced, the etching activity of the carbon matrix was enhanced to increase mesopores and macropores. Hence, the V_total_ of I, N-CNCs increased to 0.73 cm^3^/g, but the S_BET_ reduced to 640 m^2^/g. The macropores in the carbon cathodes could reduce the route of electrolyte ions approaching micropore and mesopore, effectively mitigating the diffusion resistance.

The surface functionalities and detailed elemental composition of carbon samples were analyzed by XPS, as shown in Figure 2d. The XPS survey spectrum of I, N-CNCs affirmed the signals of I and N, with the of I and N doping contents of 0.4 at.% and 7.1 at.%, respectively. The high-resolution C1s XPS spectra (Figure S3a) were separated into five peaks at 284.6 eV, 285.3 eV, 286.7 eV, 288.5 eV, and 289.3 eV, representing sp^2^-C, sp^3^-C, -C–OH/-C-N, -C=O, and -COOH groups, respectively [26]. In addition, the component ratio of sp^3^ to sp^2^ can be applied to estimate the defect degree [27]. The values of sp^3^ to sp^2^ for I, N-CNCs, and N-CNCs were 1.26, and 1.18, further confirming that more carbon defects were introduced after the iodine doping. The deconvolution high-resolution O 1s (Figure S3b) displayed the presence of oxygen-containing functional groups, corresponding to quinonyl groups (-C=O), hydroxyl group (-C-OH), ether groups (C-O-C), and carboxyl groups (-COOH) at 531.1 eV, 532.2 eV, 533.5 eV, and 535.4 eV, respectively [6]. The abundant oxygen-containing groups on the carbon surface were beneficial to the charge storage capability of Zn-ion hybrid supercapacitors. These groups increased the surface wettability between the carbon cathodes and ion electrolyte. In addition, the phenol and quinone group played a valid role in Zn^2+^ storage by reversible reaction with Zn^2+^ [28,29]. The N1s spectra of carbon samples (Figure 2e and Figure S3c) could be fitted to four different nitrogen doping groups of pyridinic N, pyrrolic N, graphitic N, and N-oxide, centered at 397.9 eV, 499.2 eV, 400.8 eV, and 403.4 eV, respectively. The pyridinic N and graphitic N accounted for the vast majority of nitrogen doping groups shared, which resulted from the maintenance of nitrogen in a triazine ring of melamine after the pyrolysis. As an electron donor, the pyridinic nitrogen possessed a larger binding energy than other N species to chemically coordinate with Zn^2+^ to provide more pseudo-capacitance [30]. The graphitic nitrogen could enhance the electronic conductivity and provide rich defects for interacting with the electrolyte [31]. The high-resolution core-level I 3d spectra of I, N-CNCs (Figure 2f) can be dissociated into two peaks of I 3d_5/2_ and I 3d_3/2_, where each peak can be further fitted into triiodide (I_3_^−^) and pentaiodide (I_5_^−^) species at the binding energies of 618.5 eV, 630.1 eV and 620.0 eV, 632.2 eV, respectively, matching with the reported I 3d XPS spectra [32,33,34].

### 3.2. Electrochemical Performance of ZHCs

**Figure 3:**
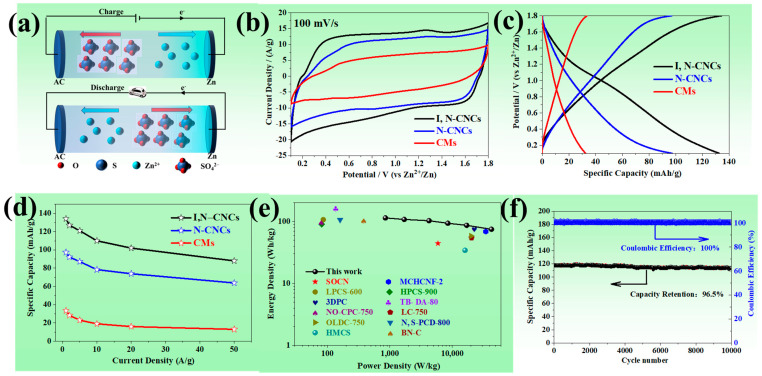
(**a**) Schematic diagram of ZHC. (**b**) CV curves at 100 mV/s, (**c**) GCD curves at 1 A/g, and (**d**) rate performance of I, N-CNCs, N-CNCs, and CMs. (**e**) Energy/power density of I, N-CNCs-based ZHCs and other reported cathode materials for ZHCs. (**f**) Cycling performance at a current density of 5 A/g of I, N-CNCs -based ZHCs.

I, N-CNCs possessed nanocages structure with cracked shells, hierarchical architecture, and rich heteroatom dopants, which resulted in senior diffusion accessibility to electrolyte ions and short transport routes for electrons. Gaining from the aforementioned superiorities, I, N-CNCs might be an effective cathode material for the ZHCs. The electrochemical performance of I, N-CNCs, N-CNCs, and CMs were then evaluated by assembling aqueous ZHCs with the obtained carbon cathodes and Zn foil anode separated by non-woven fabrics in the 2 M ZnSO_4_ electrolyte (Figure 3a). Due to the highly desirable combination between the redox-type anode and the supercapacitor-type cathode, decent energy and power densities could be gained for energy storage devices. Figure 3b displays the cyclic voltammetry (CV) profiles of the assembled supercapacitors at 100 mV/s. Among these, the peaks of water decomposition were not observed in CV curves, demonstrating that the ZHC devices can charge–discharge well from 0.1 to 1.8 V. All carbon cathodes exhibited approximate rectangular CV curves, implying the capacitance-dominated mechanism for ion storage. Additionally, the existence of evident cathodic/anionic humps at 0.8/1.3 V provided a convincing demonstration of invertible oxidation-reduction reactions from Zn^2+^ deposition/stripping on the Zn anode during the charge–discharge cycles, which generated considerable pseudo-capacity [26]. Observably, the CV curve of I, N-CNCs cathode-based ZHCs presented a larger integrated area than those of the Zn//N-CNCs and Zn//CMs in Figure 3b, revealing their highest specific capacity. In addition, the nearly rectangular CV curves of CNCs-based ZHCs could be well maintained when the scan rate was raised from 10 mV/s to 200 mV/s, while a prominent distortion to spindle shape was observed for CMs-based ZHCs at 200 mV/s, as shown in Figure S4a–c. This difference revealed the rapid ion diffusion kinetics in the CNCs electrode, which could be attributed to the interconnected cage-like structure and nanoscale diffusion distance from crack shells. The introduction of iodine enriched the adsorption sites for energy storage in the carbon nanocages and had no adverse effect on the diffusion rate of the electrolyte ion.

The electrochemical capabilities of the three ZHC devices were further researched by galvanostatic discharge–charge (GCD) tests. The GCD profiles for all supercapacitors at 1 A/g are displayed in Figure 3c. It was evident that I, N-CNCs-based devices exhibited a reversible optimum energy storage capability (134.2 mAh/g) greatly larger than those of the other two ZHCs (N-CNCs-based device 97.3 mAh/g, CMs-based device 33.5 mAh/g). This was corresponded to the results from the CV curves. In addition, the GCD profiles of three ZHCs from 1 to 50 A/g are shown in Figure S4d–f, which display symmetric triangular configurations, implying typical capacitive energy storage. The specific capacities were calculated and delineated in Figure 3d according to the corresponding GCD curves. The ZHCs of the I, N-CNCs cathode exhibited extremely satisfying capability at higher current densities, with reversible capacities of 127.7, 121.1, 110.4, and 102.6 mAh/g at 2, 5, 10, and 20 A/g, respectively. Additionally, even at a remarkable current density of 50 A/g, the Zn//I, N-CNCs still retained the invertible capacity of 88.9 mAh/g, with a capacity retention of 66.2% from the initial capacity at 1 A/g. Similarly, the Zn//N-CNCs remained at 63.8 mAh/g at the current density of 50 A/g and the corresponding capacity retention was 65.7% from the initial capacity at 1 A/g. In comparison, the capacity retention of CMs-based ZHCs at 50 A/g was only 38.8%. Generally, the energy storage capacities of Zn//I, N-CNCs and Zn//N-CNCs were superior to that of Zn//CMs. This can be ascribed to the high N content (7.1 at% and 8.7 at%) with the dominant pyridinic N (49% and 47%) from the triazine ring for I, N-CNCs and N-CNCs, respectively. The pyridinic N owned the more robust binding energy to chemically adsorb Zn^2+^ than other N species for enhanced zinc ion storage [30]. Moreover, in addition to the subscription of N heteroatoms, the structural defects (such as vacancies and edges) in carbon nanocages served as active sites for available ion storage, hence boosting electrochemical performances [35,36]. The rate performance of cage-like carbon samples (I, N-CNCs and N-CNCs) devices was significantly higher than that of the irregularity sample (CMs). Due to the big ionic dimension, the narrow micropores in CMs tend to obstruct the rapid transfer of electrolyte ions, resulting in the inferior rate performance [37]. The I, N-CNCs and N-CNCs possessed approximate specific surface area, porous structure, and N, O doping contents, but the specific capacitance of Zn//I, N-CNCs was superior to that of Zn//N-CNCs at all current densities, which verified that I doping can improve specific capacitance. Due to the relatively broad operating voltage window, decent rate capacity, and the high charge capacity, the Zn//I, N-CNCs ZHCs accomplished a maximum energy and power density of 114.1 Wh/kg and 42.5 kW/kg. Notably, the excellent energy-power output matched or even surpassed most ZHC devices reported in other studies, as exhibited in the Ragone plot (Figure 3e and Table S3). Furthermore, the long-term cycling life of the Zn//I, N-CNCs was assessed at 5 A/g, which displayed a high capacity of 119.5 mAh/g with a capacity retention of 96.5% and nearly 100% Coulombic efficiency over 10,000 cycles (Figure 3f). The mild drop in capacity was attributed to the unavoidable formation of sluggishness segments onto the Zn anode, which slowed down the ion and electron transport at interfaces [38].

**Figure 4:**
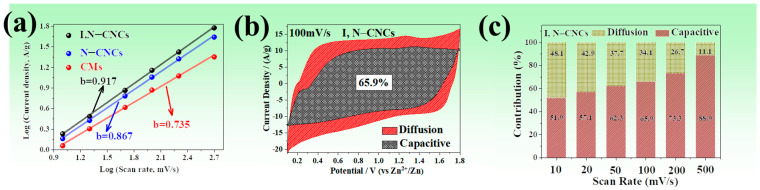
(**a**) The fitting plot between logarithmic peak current and logarithmic scan rate of the three carbon cathodes. (**b**) Capacitive contribution to the total current for I, N-CNCs-based ZHCs from CV analysis at 100 mV/s. (**c**) The capacitive and diffusion contribution ratios to the total capacity at different scan rates for I, N-CNCs-based ZHCs.

In order to understand the charge storage kinetic behaviors in the charge–discharge process, the CV curves were further analyzed by peak current (i) and sweep rate (v) according to the equation of i = av^b^ [6]. The b value was calculated by the gradient of linear log (i)-log (v). Generally, the b value of 0.5 illustrates a diffusion-controlled energy storage behavior, while the b value of 1 indicates an ideal capacitive surface-dominated energy storage behavior. As shown in Figure 4a and Figure S5, the b values of I, N-CNCs-based ZHCs and N-CNCs-based ZHCs were 0.917 and 0.878, which were larger than that of CMs-based ZHCs (0.765), indicating a larger capacitive contribution for the cage-like carbon cathodes. The contribution proportion of capacitive-dominated and diffusion-controlled behaviors were further quantitatively estimated via the following formula of i(V) = k_1_v + k_2_v^0.5^. The i(V) is the current at a constant potential. The total i can be separated into the fraction of capacitive processes current (k_1_v) and diffusion-controlled current (k_2_v^0.5^) at the potential. As demonstrated in Figure 4b and Figure S6a,b, the results revealed that I, N-CNCs and N-CNCs mainly exhibited a capacitive-controlled behavior with a proportion of 65.9% and 59.4% at 100 mV/s, higher than that of CMs (38.1%), which indicated the rapid and efficient charge storage capability of the carbon nanocages cathodes. The contributions of capacitive-controlled behavior for I, N-CNCs gradually increased from 51.9% to 88.9% of the total stored charge with increasing scan rates from 10 mV/s to 500 mV/s, maintaining a remarkably high capacitive contribution range, which surpassed the CMs cathode with only 20.6–63.6% capacitive contribution (Figure 4c and Figure S6c,d). The global analysis illustrated the co-existence of capacitance and diffusion-controlled behaviors at all scan rates, suggesting an integrated energy storage mechanism of electrical double-layer capacity and pseudo-capacity. The I, N-CNCs and N-CNCs highlighted their ability to reserve ion by chemical and physical adsorption, and facilitate electrolyte ion diffusion within the cage-like structure, especially at relatively low sweep rate. Contrastively, CMs exhibited a relatively high diffusion contribution of 79.4% at 10 mV/s, indicating that they are primarily dominated by diffusion behavior, ascribed to their largely small micropores and lack of regular structure [39]. These remarkable diversities in charge storage kinetics evidenced the superiority of hollow cage-like structures. The uncapacious micropores block up the ion diffusion to the interior pore at fast charging, leading to the inefficient utilization of active sites and sluggish transfer behavior in CMs. In comparison, the I, N-CNCs with a cage-like shell structure and rich ion-accessible active sites provided favorable pathways for electron transfer and ion diffusion, resulting in capacitive-dominated charge storage process and rapid diffusion kinetics.

### 3.3. Energy Storage Mechanism

**Figure 5:**
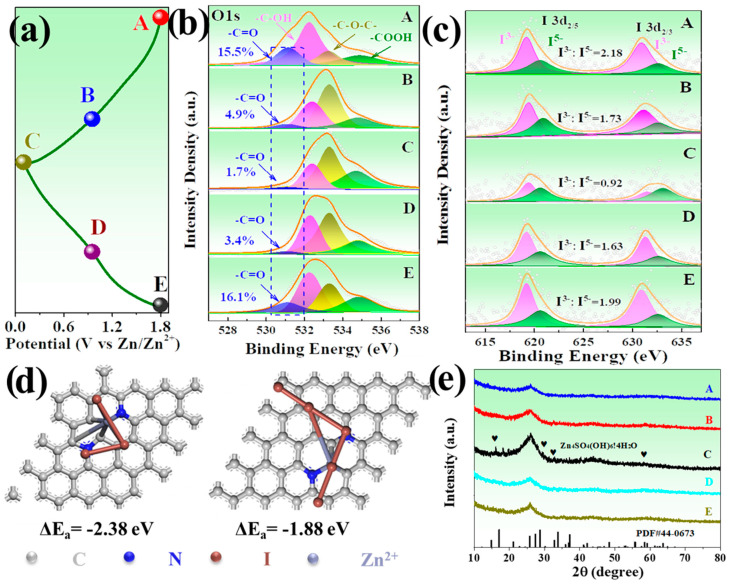
(**a**) Selected potential states points in the typical charge–discharge curves for ex-situ characterization. High-resolution (**b**) O 1s and (**c**) I 3d XPS spectra of the I, N–CNCs cathode at the selected states. (**d**) The theoretical calculation models with top–view for Zn^2+^ absorbed on the I, N–CNCs with I_3_^−^ (**left**) and I_5_^−^ (**right**) coordination around the pyridinic-N site, as well as corresponding Δ*E*_a_. (**e**) XRD patterns of the I, N–CNCs cathode at the selected states, ♥ representing Zn_4_SO_4_(OH)_6_·4H_2_O (JCPDF No. 44-0673).

To acquire a comprehensive understanding of the charge-storage process, the surface component variations in the I, N-CNCs cathode at five charging–discharging states (A: 1.8 V, B: 0.9 V, C: 0.1 V, D: 1.2 V, and E: 1.8 V) were investigated by ex situ XPS and XRD (Figure 5a). The fluctuations in Zn 2p and S 2p XPS bands were applied to account for the storage variations in Zn^2+^ and SO_4_^2−^ during the charge–discharge process. When the cathode material was discharged from 1.8V to 0.1 V (from states A to C), the gradually stronger Zn signal peaks were observed, indicating the increased Zn^2+^ cation aggregation onto the surface of the carbon cathode (Figure S7a). Subsequently, the Zn signal gradually decreased during the charging process from 0.1 V to 1.8 V (from states C to E), indicating the desorption of Zn^2+^ from the carbon cathode. Contrastively, the signal intensity of S 2p displayed an inverted variation tendency during the discharging–charging process (Figure S7b), demonstrating that the SO_4_^2−^ anion could be stored at high potential and desorbed at low potential on the surface of the carbon cathode. These processes confirmed the energy storage mechanism of the alternative absorption–desorption of anions and cations during the reversible charging/discharging processes.

The C 1s XPS spectra at five states were further studied to estimate the faradaic pseudo-capacitance between Zn ions and C-O/C=O bonds according to the fluctuations of C-O-Zn peaks in Figure S7c. The proportion of C-O-Zn peak increased from 3.2% at high potential to 9.5% at low potential during the discharge process and its percentage was again reduced to 3.5% during the charge process, proving the invertible chemisorption of Zn^2+^ with the surface oxygenic functional groups of the carbon cathode. The high-resolution O 1s XPS spectra of I, N-CNCs can be fitted into four peaks at 531.1, 532.3, 533.4, and 534.8 eV in Figure 5b, ascribing to -C=O, -C-OH, -C-O-C-, and -COOH, respectively. The content of -C=O varied from 15.5% (stage A) to 4.9% (stage B) and then achieved 1.7% (stage C) and the proportion of -C=O can be inversely retrieved to 16.1% (stage E) with the potential recovery to 1.8 V, which also demonstrated the greatly reversible pseudocapacitive energy storage via the interaction of -C=O and Zn ion [40]. The above results suggested that the oxygen functional groups effectively took part in the Zn^2+^ storage by reversible chemisorption during charge/discharge processes.

The core-level I 3d spectra of I, N-CNCs at stage A (Figure 5c) can split into two pairs of peaks located at 619.1/630.8 eV and 620.6/632.7 eV, which were be assigned to the I_5_^−^ (I 3d_5/2_) and I_3_^−^ (I 3d_3/2_) of the C^+^-I_5_^−^ and C^+^-I_3_^−^ bonds [33]. The two pairs of peaks shifted to slightly higher binding energies by ∼0.6 eV relative to that in the initial state (Figure 2f). Probably, this shift was attributed to the chemisorption of Zn^2+^ with the I_5_^−^/I_3_^−^ of the carbon cathode. The I_5_^−^/I_3_^−^ as a nucleophile had a nucleophilicity to preferentially adsorb the Zn^2+^ by the formation of C^+^-I_5_^−^-Zn^2+^ and C^+^-I_3_^−^-Zn^2+^, which had an influence on the intensities and binding energies of I 3d spectra [41]. With the continuous discharge, the peaks of the C^+^-I_5_^−^ and C^+^-I_3_^−^ bonds further shifted toward the higher binding energies at stage C. Simultaneously, these peaks’ intensities underwent a decrease (A to C). When the device came to the charge process from stage C to stage E, the peak positions of C^+^-I_5_^−^ and C^+^-I_3_^−^ bonds could be inversely retrieved to the binding energies of stage A and the intensities of corresponding peaks were recovered. The signal variation in C^+^-I_5_^−^ and C^+^-I_3_^−^ bonds in strength and position confirmed that the I_5_^−^/I_3_^−^ groups effectively participated in the Zn^2+^ storage by invertible chemisorption during charge–discharge processes. The iodine has an admirable combination with zinc to store energy, which has been also demonstrated in other promising energy-storage systems [11]. Concurrently, the ratio of I_5_^−^/I_3_^−^ reduced from 2.18 (stage A) to 1.73 (stage B) and then achieved 0.92 (stage C) during the discharge process and the proportion of I_5_^−^/I_3_^−^ can be gradually recovered to 1.99 (stage E) with the potential recovering to 1.8 V, implying that the C^+^-I_5_^−^ and C^+^-I_3_^−^ groups undergo reversible variation during charge/discharge processes. This meant that the intensity variations in I_3_^−^ appeared more obvious that in I_5_^−^, which implied that the Zn^2+^ preferentially bonds with I_3_^−^ rather than I_5_^−^.

To better understand how iodine doping affects the adsorption of Zn^2+^ on the surface of carbon cathodes, DFT calculations were carried out to estimate the adsorption energy (ΔE_a_) between Zn^2+^ and C^+^-I_3_^−^/C^+^-I_5_^−^. For the convenience of DFT calculations, the I_3_, N-doped graphene and I_5_, N-doped graphene (Figure 5d) were chosen as contrastive models to research their interactions with Zn^2+^ ions. The Zn^2+^ ion could be chemically adsorbed in the nitrogen and iodine with binding energies of −2.38 eV and −1.88 eV for I_3_^−^, N-doped graphene and I_5_, N-doped graphene, respectively, as a result of their donation of electrons to the unoccupied orbital of Zn^2+^ ions [30,42]. Importantly, I_3_^−^ was confirmed to better facilitate Zn^2+^ storage by chemical coordination with the more negative binding energy, which was consistent with the analytical conclusions from XPS.

Figure S8 displayed the SEM images of I, N-CNCs at different stages over discharge–charge processes. When the carbon cathode was discharged from state A to state C, a small amount of nanosheet-like zinc hydroxide sulfate hydrate nanosheets emerged among the carbon nanocages, which were marked in red circles. As the potential recovered to 1.8 V (state E), the nanosheets faded out, but the carbon morphology maintained structural stability. According to the XRD patterns in Figure 5e, the nanosheets were identified as Zn_4_SO_4_(OH)_6_·4H_2_O (JCPDF No. 44-0673). When the device was discharged to 0.1 V (state C), the characteristic peaks of the Zn_4_SO_4_(OH)_6_·4H_2_O were observed at 17.1°, 28.6°, 33.8°, and 58.4° corresponding to the (0 0 4), (−1 0 6), (3 −1 2), and (4 1 0) lattice planes. When the device was recharged to the high potential 1.8 V (state E), the above characteristic peaks became faint (Figure 5e). The variation implied that the appearance and disappearance of zinc hydroxide sulfate hydrate may have contributed to the partly reversible energy storage. The precipitation of zinc hydroxide sulfate hydrate suggested that OH- may participated in the energy storage reaction.

**Figure 6:**
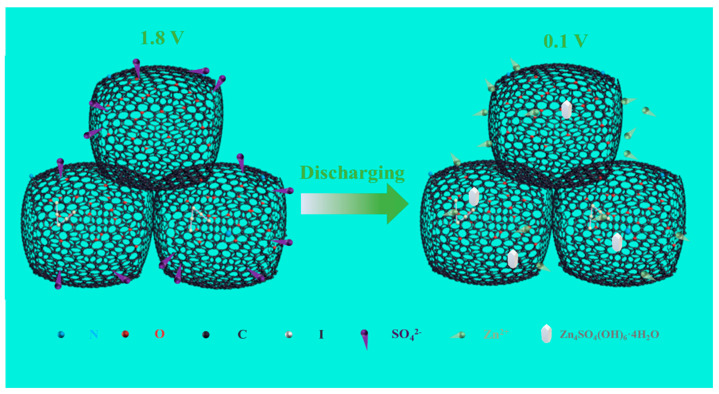
Schematic diagram of the charge storage mechanism of the I, N—CNCs cathode.

According to the conclusion of the experiments and the above systematic analysis, the cathode charge storage mechanism of I, N-CNCs-based ZHCs can be diagrammatically illustrated in Figure 6. First, the SO_4_^2−^ anion and Zn^2+^ cation primarily alternate adsorption mechanisms, which was confirmed during charge/discharge processes. During the discharge process, SO_4_^2−^ anions were desorbed from the carbon nanocages in high-potential regions, while the absorption of Zn^2+^ cation happened in low-potential regions with accumulation onto the surface of carbon nanocages. The ions adsorption and desorption process were reversed during the discharge process. In addition, the Zn^2+^ ion was bound with C=O and C^+^-I_5_^−^/C^+^-I_3_^−^ by chemical adsorption with additional pseudo-capacitance. Moreover, the Zn_4_SO_4_(OH)_6_·4H_2_O partial precipitation and dissolution were attributed to the variation in pH from H^+^ adsorption/desorption [26]. The OH^−^ reacted with the Zn^2+^ and SO_4_^2−^ to prepare zinc hydroxide sulphate hydrate on the cathode surface with the increase in concentration, which was an important contribution for the Zn^2+^ storage.

To evaluate the practical energy storage requirements for energy storage devices, the quasi-solid-state supercapacitor was prepared by applying Zn foil as the anode, I, N-CNCs as the cathode, and ZnSO_4_/gelatin gel as an electrolyte. The CV curves at Figure S9a displayed the energy storage process of a quasi-solid-state device, which was similar to that of the aqueous device, indicating satisfactory operation of the supercapacitor in the ZnSO_4_/gelatin gel electrolyte. Moreover, the working voltage was broadened to 5.1 V when three ZHCs were connected in series and the capacity of three ZHCs linked in parallel increased three times that of a single ZHC. To project the utility performance, ten purple LEDs could be energized by three quasi-solid-state supercapacitors in series, as shown in Figure S9b. Therefore, the assembled quasi-solid Zn-ion hybrid supercapacitors devices possessed a promising potential in renewable energy storage.

## 4. Conclusions

In this work, we reported a self-assembly template-free strategy to prepare nanocage-like carbon cathodes for ZHCs with a strong hydrogen-bonding interaction derived from ellagic acid, with the capability to participate in hydrogen bonding and π-π coactions, and melamine. The large-scale production of iodine-doped carbon nanocages would be an environmentally friendly strategy and a scalable approach suitable for practical applications, which overcomes a great deal of challenges, such as tedious synthetic procedures, low efficiency, and environmental concerns caused by etching agents. The charge-storage processes by ex situ XPS to analyze surface component variations of the I, N-CNCs cathode confirmed that the I_5_^−^/I_3_^−^ groups effectively participated in the Zn^2+^ storage by invertible chemisorption during charge–discharge processes. DFT calculations were carried out to examine the adsorption energy between Zn^2+^ and C^+^-I_3_^−^/C^+^-I_5_^−^. Importantly, I_3_^−^ was confirmed to better facilitate Zn^2+^ storage by chemical coordination with the more negative binding energy. Therefore, the optimized I, N-CNCs carbon cathode exhibited excellent capacity (134.2 mAh/g), and remarkable energy density (141.1 Wh/kg) and power density (42.5 kW/kg), along with super-long cycle stability (96.5% capacity retention after 10,000 cycles). These impressive results provide a perspective for the facile synthesis of cage-like carbon materials to enrich the research scope of energy storage and catalysis application and elucidate the mechanism of electron donor dopants for boosting Zn-ion storage.

## Data Availability

Data are contained within the article.

## Supplementary Materials

The following supporting information can be downloaded at: https://www.mdpi.com/article/10.3390/ma17040838/s1, Figure S1: TEM images of (a) hydrothermal melamine, ellagic acid, and ammoni. Figure S2. The corresponding EDS spectrum of nanocages and EDS elemental mapping images of C, N, O, and I. Figure S3: (a) C 1s, (b) O 1s and (c) N 1s high-resolution XPS spectra of N-CNCs and I, N-CNCs. Figure S4: Cyclic voltammetry curves of (a) I, N-CNCs, (b) N-CNCs and (c) CMs based zinc ion capacitors at scan rates range of 10–200 mV/s. Galvanostatic charge-discharge profiles at 1–50 A/g for (d) I, N-CNCs, (e) N-CNCs and (f) CMs based zinc ion capacitors. Figure S5: The fitting plot between logarithmic peak current and logarithmic scan rate for (a) I, N-CNCs, (b) N-CNCs and (c) CMs. Figure S6: Capacitive contribution ratios from CV analysis at 100 mV/s of (a) N-CNCs (b) CMs based ZHC. The capacitive and diffusion contribution ratios to the total capacity at different scan rates for (c) N-CNCs (d) CMs based ZHC. Figure S7: Ex-situ XPS of high-resolution (a) Zn 2p, (b) S 2p, and (c) C 1s XPS spectra. Figure S8. Ex-situ SEM images of I, N-CNCs at various voltage points at five states, A: 1.8 V, B: 0.9 V, C: 0.1 V, D: 1.2 V and E: 1.8 V. The Zn4SO4(OH)6·4H2O nanosheets were marked in red circles. Figure S9: (a) CV curves of quasi-solid-state ZHC at various scan rates. (b) CV curves of three devices in series and in parallel at 100 mV/s, background: purple blue LEDs powered by three devices in series. Table S1 Lattice parameters of carbon materials samples. Table S2: Pore parameters of carbon materials samples. Table S3: Energy/power density of I, N-CNCs and other reported cathode materials for Zinc ion hybrid capacitors. References [26,38,43,44,45,46,47,48,49,50,51,52] are cited in the supplementary materials.
