# Supplementary material for: Iodine-Doped Hollow Carbon Nanocages without Templates Strategy for Boosting Zinc-Ion Storage by Nucleophilicity
Source: Materials (Basel). 2024 Feb 9;17(4):838. doi: 10.3390/ma17040838 (PMC10890013; doi:10.3390/ma17040838)
Supplement: Supplementary file 1 [file materials-17-00838-s001.zip › materials-2850894-supplementary.pdf]

Supporting Information

Iodine-Doped Hollow Carbon Nanocages without Templates Strategy for Boosting Zinc-Ion Storage by Nucleophilicity

Ruiting Niu [1], Huailin Fan [1,*], Qingfu Ban [2], Dezhi Zhou [1], Lekang Zhao [1], Jiayuan Yu [3,*], Qifeng Chen [1,*] and Xun Hu [1,*]

[1] School of Material Science and Engineering, University of Jinan, Jinan 250022, China
[2] College of Chemistry and Chemical Engineering, Yantai University, 30 Qingquan Road, Yantai 264005, China
[3] Institute for Advanced Interdisciplinary Research (iAIR), School of Chemistry and Chemical Engineering, University of Jinan, Jinan 250022, China
* Correspondence: huailinfan@163.com (H.F.); ifc_yujy@ujn.edu.cn (J.Y.); mse_chenqf@ujn.edu.cn (Q.C.); xun.hu@outlook.com (X.H.)

Synthesis of the carbon nanocages

2 g melamine, 0.5 g ammonium iodide and 2 g ellagic acid were dispersed in 60 mL distilled water under stirring for 1 h. The mixture was transferred into a Teflon-lined stainless-steel autoclave and further kept 180 °C for 16 h. The solid product was collected by filtration after cooled down to room temperature. After this, the collected hybrids were directly carbonized at 900 °C for 1 h in N2 atmosphere at heating rate of 5 °C/min. The obtained carbon product was also further activated with potassium hydroxide (mass ratio: 1:1) at 900 °C for 1 h. The activated sample was washed with 0.5 M hydrochloric acid and dried at 70 °C, termed as I, N-CNCs. For comparison, 0.5 g ammonium iodide and 2 g ellagic acid was hydrothermal treated and activated as N-CNCs (activated carbon spheres). CMs was prepared with 2 g ellagic acid with the same condition of I, N-CNCs.

Morphological and Structural Characterization

The microstructures of the samples were investigated in a Regulus 8100 SEM manipulated at 5 kV (Hitachi High-Technologies Corporation) and transmission electron microscope (TEM, JEM-2100 Plus) with a field emission gun operating at 200 kV. The crystal structure was characterized by powder XRD (Ultima IV X–ray, Japan) with a Cu target (Kα–radiation source, λ = 1.5406 Å). X-ray photoelectron spectroscopy (XPS) spectra were recorded on a Perkin-Elmer PHI pectrometer with monochromatic Al Kα (1486.6 eV) source by ESCALAB 250 (Thermo Electron). Nitrogen adsorption-desorption experiments were conducted in an ASAP 2020 instrument at 77 K. Raman measurements were performed on a LabRAM HR Evolution (France) with a laser wavelength of 532 nm.

Electrochemical Measurements

Aqueous ZHCs devices were assembled with carbon cathode and Zn foil anode/current collector separated by Whatman filter paper separator and 2.0 M ZnSO4 aqueous electrolyte in a homemade mould. A slurry carbon cathode was obtained with active material (80 wt%), acetylene black (10 wt%) and poly(vinylidene fluoride) binder (10 wt%). The resulting slurry was then brush-coated on one side of a carbon paper current collector drying at 120 °C for 6 h. Total mass loading of the electrode was ~2 mg/cm2. Cyclic voltamogram and galvanostatic charge-discharge curves were obtained by CHI 760E, Shanghai Chenhua, China. The ZHCs cycle testing was performed in room environment

on a LAND CT2001A instrument.

The gelatin (3 g) was dissolved in $ZnSO_4$ solution (15 mL, 1 mol/L) with continuous stirring at 60 ^{0}C. After becoming clear, the mixed solution was poured into a glass mold and kept at ~4 ^{0}C in a refrigerator for 12 h. Then, quasi-solid-state electrolyte films with a typical thickness of 1 mm were obtained and cut into pieces with desirable sizes for subsequent ZHCs assembly. Quasi-solid-state ZHCs devices were assembled with gelatin/$ZnSO_4$ gel as a separator. The quasi-solid device was further sealed with polyimide tape.

Theoretical Calculations

The calculations were carried out with density functional theory (DFT) in the Castep module of Materials Studio to calculate the energy of different systems. The geometry of the material was optimized first, and then system energy was calculated. To achieve structure convergence, Perdew-Burke-Ernzerhof (PBE) exchange-correlation function was performed, and the systematic energy calculation was carried out on the bases of the generalized gradient approximation (GGA). In the calculation process, the energy cutoff, the quality of the k-points, SCF tolerance, pseudopotentials were set as 500 eV, ultra-fine, 5.0e−6, and ultrasoft. The parameters of maximum force, maximum stress, and maximum displacement are set as 0.01 eV/Å, 0.02 GPa, and 5.0e−4, respectively.

Calculation

(1) The Trasatti and Dunn analysis:

$$i(V)=k_1v + k_2v^{1/2}$$

Where i (V) was the measured current as a function of potential that was considered to be consisted of capacitive currents (which vary as k_1v) and diffusion-controlled currents (which vary as $k_2v^{1/2}$).

$$i(V)/v^{1/2} = k_1v^{1/2} + k_2$$

Based on the Equation, we can obtain the values of k_1 and k_2 by plotting the relationship curves of $i/v^{1/2}$ and $v^{1/2}$, and thus both charge storage contributions are evaluated.

(2) The specific capacities (Cm mAh/g) were calculated from the GCD curves using the following equations:

$$C_m = \frac{2\,I\int V\mathrm{dt}}{3.6Vm}$$

Where I (A), $\int$ Vdt (Vs), V (V) and m (g) represent the discharge current (A), the integral area under discharge curve, the voltage after ohmic drop and m was the mass loading.

(3) Mass energy density E and mass power density P were obtained from the following equations:

$$\mathrm{E} = \frac{I\int V\mathrm{dt}}{3.6m}$$

$$\mathrm{P} = \frac{3600E}{t}$$

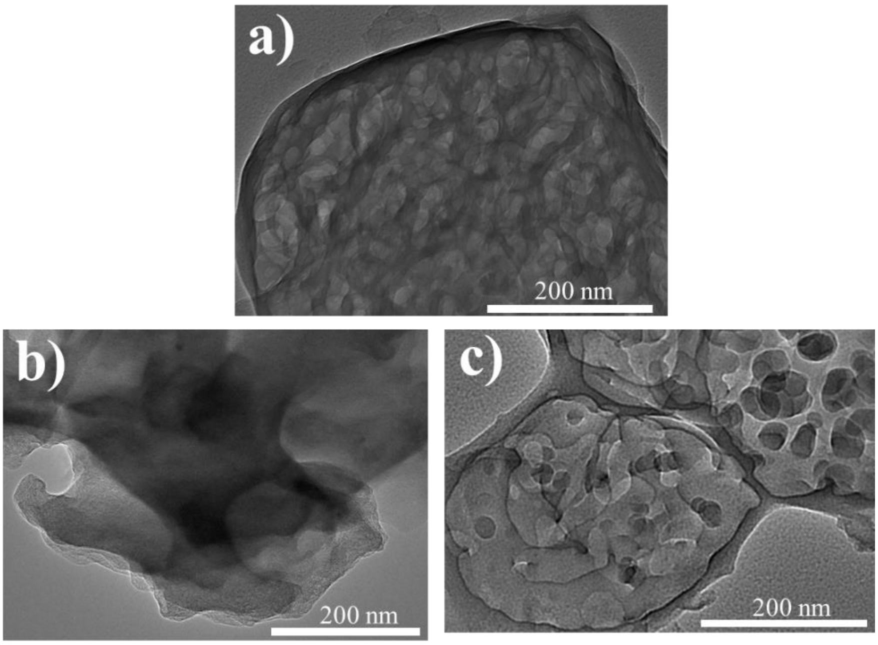

Figure S1. TEM images of a) hydrothermal melamine, ellagic acid, and ammonium iodide, b) hydrothermal ellagic acid, c) hydrothermal ellagic acid and melamine.

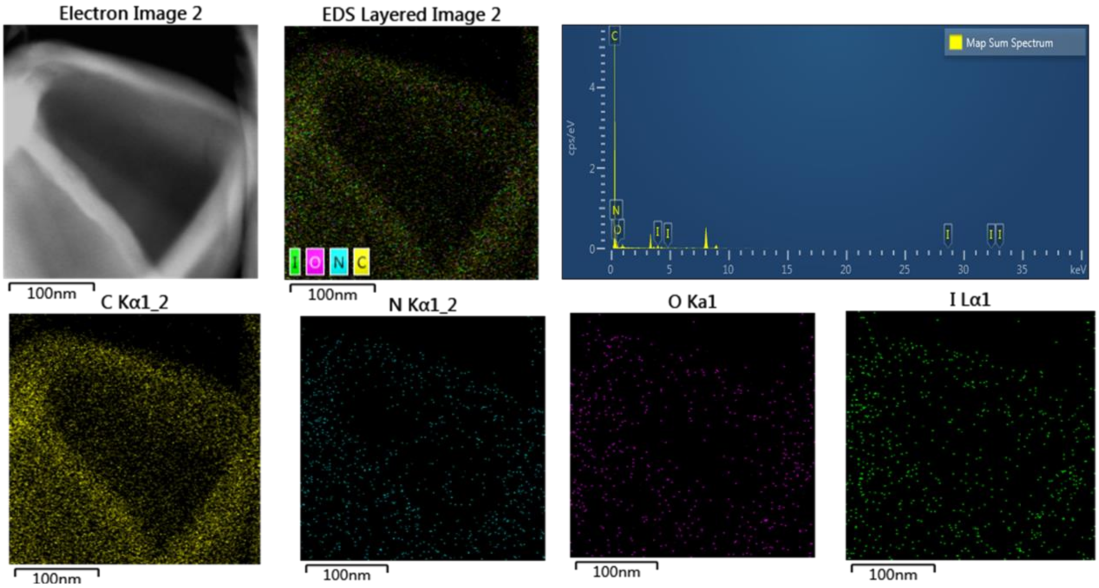

Figure S2. The corresponding EDS spectrum of nanocages and EDS elemental mapping images of C, N, O, and I.

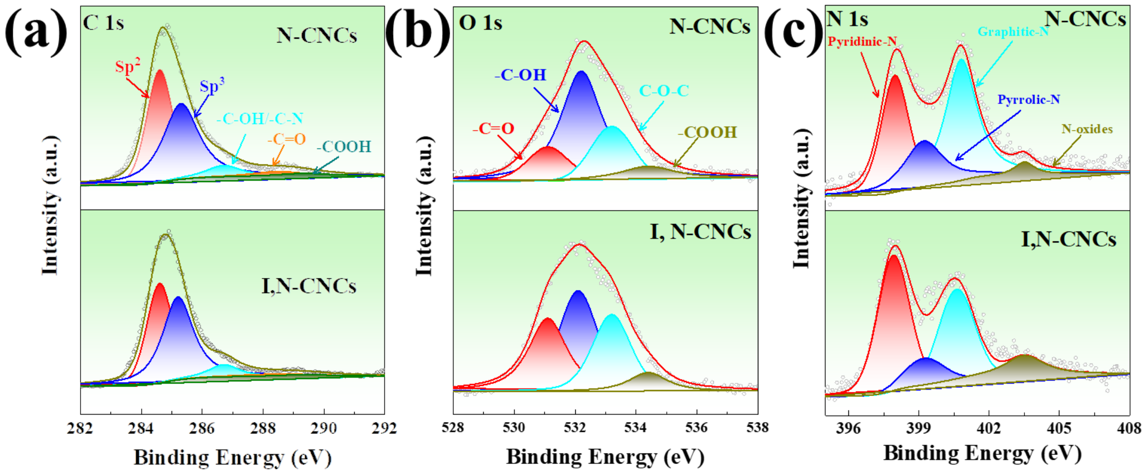

Figure S3. a) C 1s, b) O 1s and c) N 1s high-resolution XPS spectra of N-CNCs and I, N-CNCs.

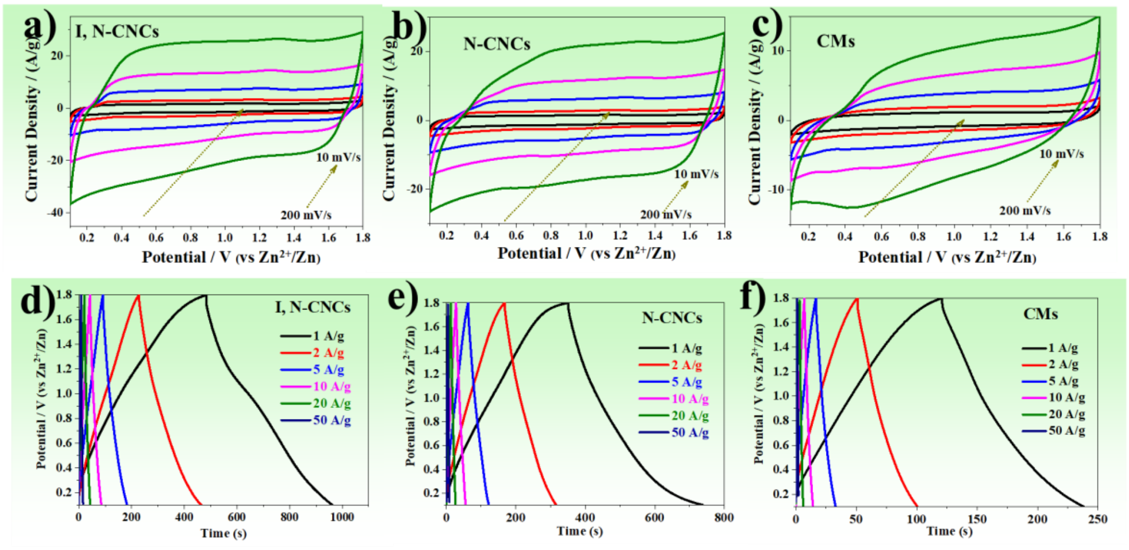

Figure S4. Cyclic voltammetry curves of a) I, N-CNCs, b) N-CNCs and c) CMs based zinc ion capacitors at scan rates range of 10-200 mV/s. Galvanostatic charge-discharge profiles at 1-50 A/g for d) I, N-CNCs, e) N-CNCs and f) CMs based zinc ion capacitors.

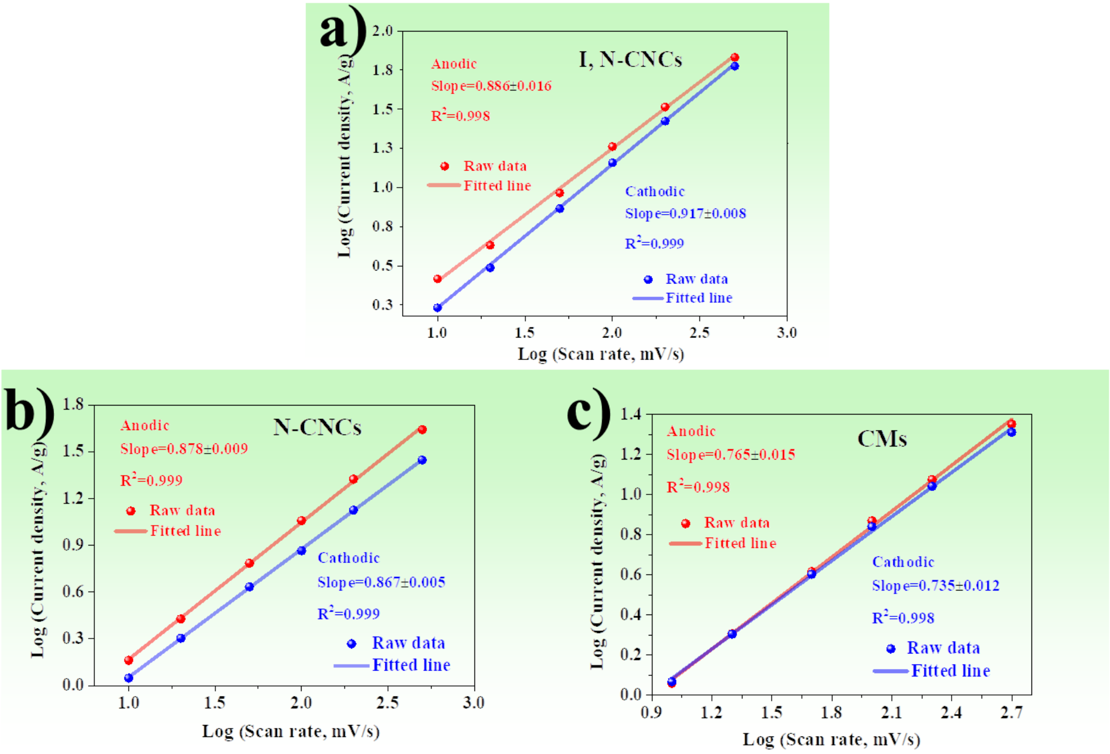

Figure S5. The fitting plot between logarithmic peak current and logarithmic scan rate for a) I, N-CNCs, b) N-CNCs and c) CMs.

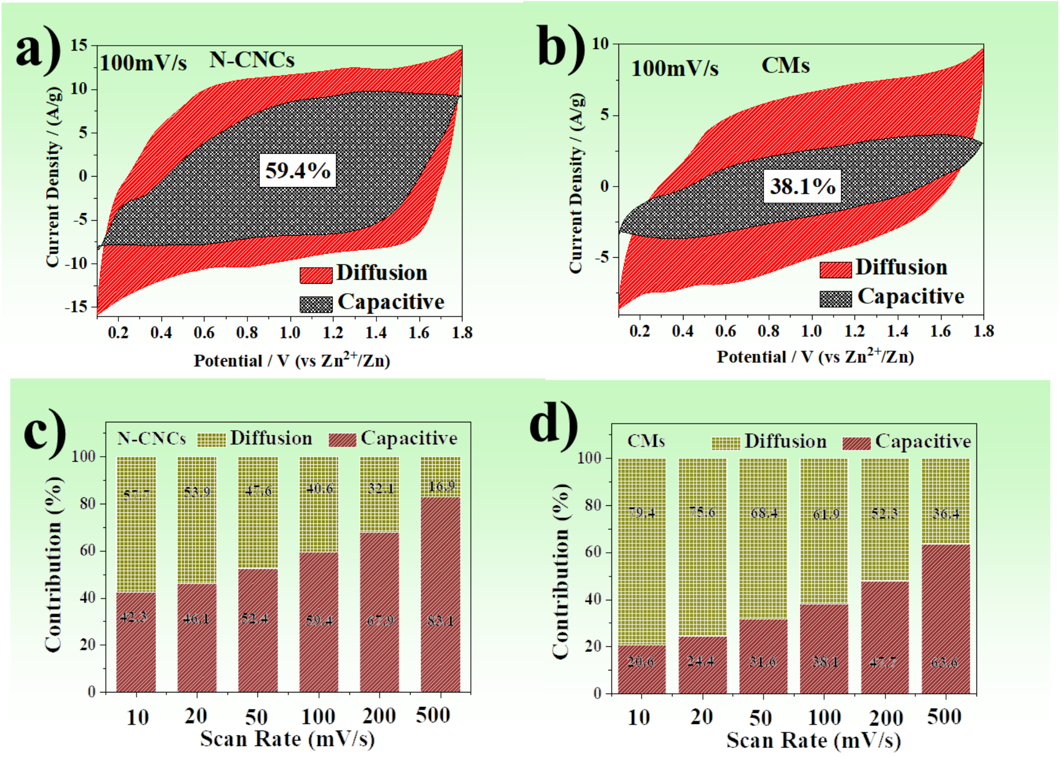

Figure S6. Capacitive contribution ratios from CV analysis at 100 mV/s of a) N-CNCs b) CMs based ZHC. The capacitive and diffusion contribution ratios to the total capacity at different scan rates for c) N-CNCs d) CMs based ZHC.

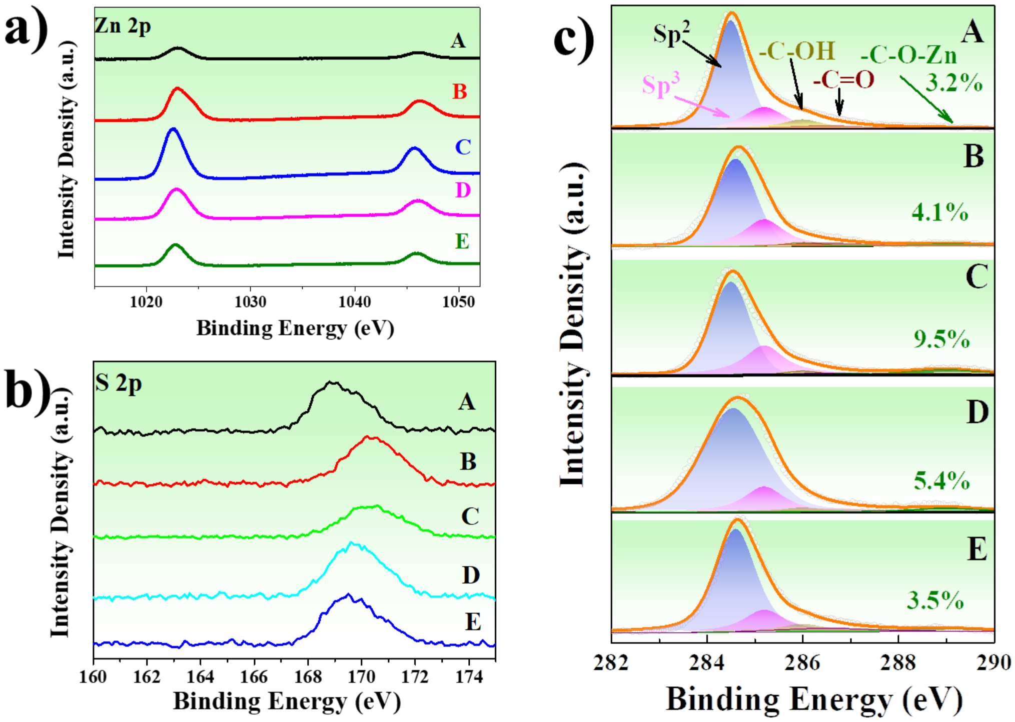

Figure S7. Ex-situ XPS of high-resolution a) Zn 2p, b) S 2p, and c) C 1s XPS spectra.

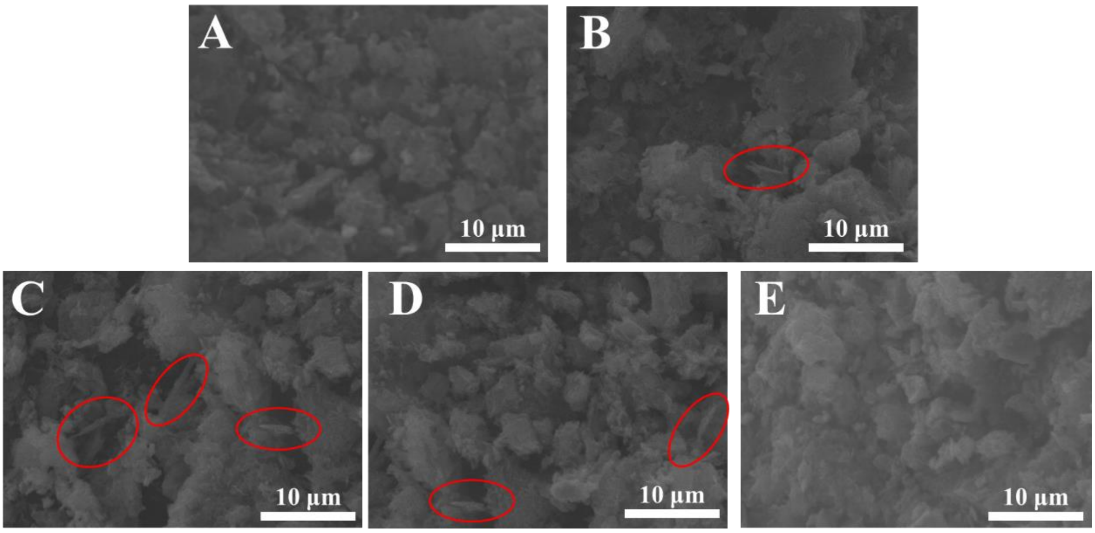

Figure S8. Ex-situ SEM images of I, N-CNCs at various voltage points at five states, A: 1.8 V, B: 0.9 V, C: 0.1 V, D: 1.2 V and E: 1.8 V . The $Zn_4SO_4(OH)_6{\cdot}4H_2O$ nanosheets were marked in red circles.

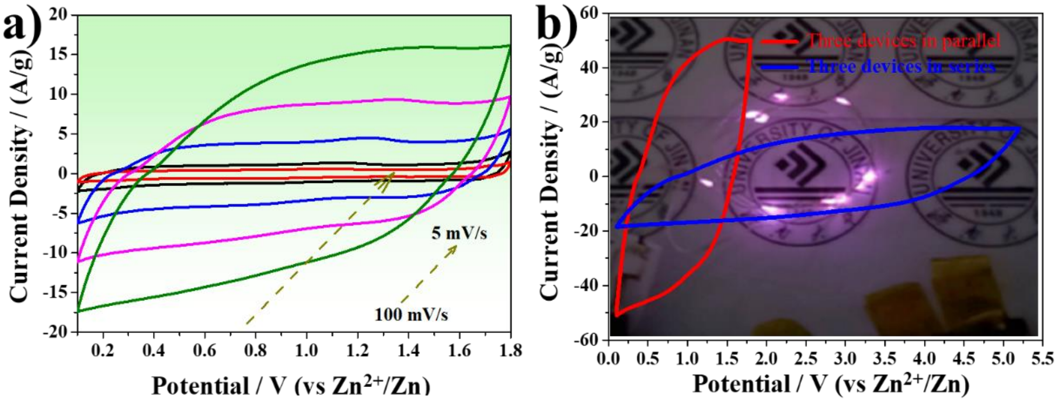

Figure S9. (a) CV curves of quasi-solid-state ZHC at various scan rates. (b) CV curves of three devices in series and in parallel at 100 mV/s, background: purple blue LEDs powered by three devices in series.

Table S1 Lattice parameters of carbon materials samples.

Sample	2θ (°)	d_{002} * (nm)	L_c ** (nm)	L_a *** (nm)
I, N-CNCs	25.9	0.35	1.6	20.1
N-CNCs	25.9	0.35	1.9	20.3
CMs	24.7	0.36	1.1	19.1

* d_{002} is calculated following Bragg's diffraction equation: 2dsinθ = nλ, where n = 1, and λ = 0.154 nm.

** L_c is calculated based on Scherrer's equation: D = Kλ/(βcosθ). In this study, D is L_c, K is a constant of 0.89, β is full width at haft maximum (FWHM), and λ = 0.154 nm.

*** L_a is calculated based on Tuinstra and Koenig equation: $L_a = (2.4 \times 10^{-10})\ \lambda_{nm}^4\ (I_G/I_D)$. In this study, λ_{nm} is the laser wavelength of 532 nm.

Table S2 Pore parameters of carbon materials samples.

Sample	S_{BET} (m^2/g)	V_{total} (cm^3/g)	V_{micro} (cm^3/g)	$V_{meso+macro}$ (cm^3/g)	V_{micro}/V_{total}
I, N-CNCs	640	0.73	0.25	0.48	0.34
N-CNCs	813	0.59	0.28	0.31	0.47
CMs	944	0.42	0.35	0.07	0.83

Table S3 Energy/power density of I, N-CNCs and other reported cathode materials for Zinc ion hybrid capacitors.

Cathode	E/Wh kg^{-1}	P/W kg^{-1}	Ref.
I, N-CNCs	114.07	850	This Work
	74.8	42500	
SOCN	44.2	5896.3	[1]
MCHCNF-2	68.7	34400	[2]
LPCS-600	107	86	[3]
HPCS-900	90.2	81.2	[4]
3DPC	76	22400	[5]
TB- DA-80	163	138	[6]
NO-CPC-750	97.8	80	[7]
LC-750	54.6	20300	[8]
OLDC-750	58.3	20000	[9]
N, S-PCD-800	106.7	160	[10]
HMCS	34.2	16000	[11]
BN-C	105.1	382.6	[12]

References

1. Zhu, C.; Long, R.; Zhu, L.; Zou, W.; Zhang, Y.; Gao, Z.; Shi, J.; Tian, W.; Wu, J.; Wang, H. Sulfate template induced S/O doped carbon nanosheets enabling rich physi/chemi-sorption sites for high-performance zinc ion hybrid capacitors. *J. Colloid. Interface Sci.* **2023**, *652*, 590–598.
2. Zhang, Y.; Zhu, C.; Xiong, Y.; Gao, Z.; Hu, W.; Shi, J.; Chen, J.; Tian, W.; Wu, J.; Huang, M.; et al. Multi-Channel Hollow Carbon Nanofibers with Graphene-Like Shell-Structure and Ultrahigh Surface Area for High-Performance Zn-Ion Hybrid Capacitors. *Small Methods* **2023**, *7*, e2300714.
3. Wen, F.; Yan, Y.; Sun, S.; Li, X.; He, X.; Meng, Q.; Zhe Liu, J.; Qiu, X.; Zhang, W. Synergistic effect of nitrogen and oxygen dopants in 3D hierarchical porous carbon cathodes for ultra-fast zinc ion hybrid supercapacitors. *J. Colloid. Interface Sci.* **2023**, *640*, 1029–1039.
4. Shang, K.; Liu, Y.; Cai, P.; Li, K.; Wen, Z. N, P, and S co-doped 3D porous carbon-architectured cathode for high-performance Zn-ion hybrid capacitors. *J. Mater. Chem. A* **2022**, *10*, 6489–6498.
5. Wang, Y.; Yang, J.; Liu, S.; Che, X.; He, S.; Liu, Z.; Wang, M.; Wang, X.; Qiu, J. 3D graphene-like oxygen and sulfur-doped porous carbon nanosheets with multilevel ion channels for high-performance aqueous Zn-ion storage. *Carbon.* **2023**, *201*, 624–632.
6. Qin, Y.; Song, Z.; Miao, L.; Hu, C.; Chen, Y.; Liu, P.; Lv, Y.; Gan, L.; Liu, M. Hydrogen-bond-mediated micelle aggregating self-assembly towards carbon nanofiber networks for high-energy and long-life zinc ion capacitors. *Chem. Eng. J.* **2023**, *470*, 144256.
7. Li, X.; Hu, J.; Wu, M.; Guo, C.; Bai, L.; Li, J.; Li, Y.; Luo, D.; Duan, J.; Li, X.; et al. Fabrication and morphological effect of waxberry-like carbon for high-performance aqueous zinc-ion electrochemical storage. *Carbon.* **2023**, *205*, 226–235.
8. Li, H.X.; Shi, W.J.; Liu, L.Y.; Zhang, X.; Zhang, P.F.; Zhai, Y.J.; Wang, Z.Y.; Liu, Y. Fabrication of dual heteroatom-doped graphitic carbon from waste sponge with "killing two birds with one stone" strategy for advanced aqueous zinc-ion hybrid capacitors. *J. Colloid. Interface Sci.* **2023**, *647*, 306–317.
9. Li, H.; Su, P.; Liao, Q.; Liu, Y.; Li, Y.; Niu, X.; Liu, X.; Wang, K. Olive Leaves-Derived Hierarchical Porous Carbon as Cathode Material for Anti-Self-Discharge Zinc-Ion Hybrid Capacitor. *Small* **2023**, *19*, e2304172.
10. Yang, Y.; Chen, D.; Wang, H.; Ye, P.; Ping, Z.; Ning, J.; Zhong, Y.; Hu, Y. Two-step nitrogen and sulfur doping in porous carbon dodecahedra for Zn-ion hybrid supercapacitors with long term stability. *Chem. Eng. J.* **2022**, *431*, 133250.
11. Chen, S.; Yang, G.; Zhao, X.; Wang, N.; Luo, T.; Chen, X.; Wu, T.; Jiang, S.; van Aken, P.A.; Qu, S.; et al. Hollow Mesoporous Carbon Spheres for High Performance Symmetrical and Aqueous Zinc-Ion Hybrid Supercapacitor. *Front. Chem.* **2020**, *8*, 663.
12. Zhu, C.-L.; Wang, H.-L.; Fan, W.-J.; Zhai, S.-L.; Wang, X.-J.; Shi, J.; Huang, M.-H.; Liu, S.; Li, Z.; Chen, J.-W. Large-scale doping-engineering enables boron/nitrogen dual-doped porous carbon for high-performance zinc ion capacitors. *Rare Met.* **2022**, *41*, 2505–2516.
